# Correction: Association between Oral Health Status and Type 2 Diabetes Mellitus among Sudanese Adults: A Matched Case-Control Study

**DOI:** 10.1371/journal.pone.0094575

**Published:** 2014-04-02

**Authors:** The PLOS ONE Staff

**Notice of Republication**

This article was republished on March 17, 2014, to replace an incorrectly published version. In the original article, the signs (≥ , ≤ , <, >) did not appear correctly throughout the text. Please download this article again to view the correct version. The originally published, uncorrected article and the republished, corrected article are provided here for reference.

## Supporting Information

File S1 **Originally published, uncorrected article.**
(PDF)

File S2 **Republished corrected article.**
(PDF)
