# Supplementary material for: Correction: Association between Oral Health Status and Type 2 Diabetes Mellitus among Sudanese Adults: A Matched Case-Control Study
Source: PLoS One. 2014 Apr 2;9(4):e94575. doi: 10.1371/journal.pone.0094575 (PMC3973585; doi:10.1371/journal.pone.0094575)
Supplement: File S2 — Republished corrected article. (PDF) [file pone.0094575.s002.pdf]

OPEN ACCESS Freely available online

PLOS ONE

Association between Oral Health Status and Type 2 Diabetes Mellitus among Sudanese Adults: A Matched Case-Control Study

Hasaan G. Mohamed[1,2]*, Shaza B. Idris[1], Mutaz F. Ahmed[3], Olav E. Bøe[1], Kamal Mustafa[1], Salah O. Ibrahim[1], Anne N. Åstrøm[1]

1 Department of Clinical Dentistry, Faculty of Medicine and Dentistry, University of Bergen, Bergen, Norway, **2** Department of Oral Rehabilitation, Faculty of Dentistry, University of Khartoum, Khartoum, Sudan, **3** Senior Consultant in Endodontics/Restorative Dentistry, Hamad Medical Corporation, Doha, Qatar

Abstract

Aim: The purpose of this study was to compare the clinical and subjective oral health indicators of type 2 diabetic patients (T2DM) with age and gender matched non-diabetic controls. A second aim was to identify clinical and subjective oral health indicators that discriminate between well-controlled and poorly controlled T2DM patients as well as between patients with long and short duration of the disease.

Methods: A total of 457 individuals participated in the study (154 T2DM cases and 303 non-diabetic controls). The T2DM group was sub-divided according to metabolic control [(well-controlled: glycosylated haemoglobin test ≤8%), (poorly controlled: glycosylated haemoglobin test > 8%)] and according to duration of T2DM [(long duration: >10 years), (short duration: ≤10 years)]. Participants were interviewed using a structured questionnaire including socio-demographics, lifestyle and oral health related quality of life factors. The clinical examination comprised full mouth probing depths, plaque index, tooth mobility index, furcation involvement and coronal and root surface caries.

Results: The T2DM patients presented with more probing depths ≥4mm, furcation involvement, tooth mobility, missing teeth, and oral impacts on daily performance (OIDP). The corresponding adjusted odds ratios and their 95% confidence intervals were 4.07 (1.74–9.49), 2.96 (1.36–6.45), 5.90 (2.26–15.39), 0.23 (0.08–0.63) and 3.46 (1.61–7.42), respectively. Moreover, the odds ratio was 2.60 (1.21–5.55) for the poorly controlled T2DM patients to have high levels of mobility index and 2.94 (1.24–6.94) for those with long duration of T2DM to have high decayed, missed and filled teeth (DMFT) values.

Conclusion: This study revealed that chronic periodontitis, tooth mobility, furcation involvement and OIDP were more prevalent among T2DM patients compared to their non-diabetic controls.

Citation: Mohamed HG, Idris SB, Ahmed MF, Bøe OE, Mustafa K, et al. (2013) Association between Oral Health Status and Type 2 Diabetes Mellitus among Sudanese Adults: A Matched Case-Control Study. PLoS ONE 8(12): e82158. doi:10.1371/journal.pone.0082158

Editor: Irina Kerkis, Instituto Butantan, Brazil

Received August 31, 2013; **Accepted** October 21, 2013; **Published** December 11, 2013

Copyright: © 2013 Mohamed et al. This is an open-access article distributed under the terms of the Creative Commons Attribution License, which permits unrestricted use, distribution, and reproduction in any medium, provided the original author and source are credited.

Funding: This study was supported by the Norwegian State Educational Loan Fund (Quota Programme). The funders had no role in study design, data collection and analysis, decision to publish, or preparation of the manuscript.

Competing Interests: The authors have declared that no competing interests exist.

* E-mail: hasaan.mohamed@student.uib.no

Introduction

Diabetes Mellitus (DM) is a metabolic disorder characterized by chronic hyperglycaemia and disturbances of carbohydrate, fat and protein metabolism [1]. Type 1 DM is most common in children and adolescents, whereas type 2 DM (T2DM) affects adults. T2DM constitutes about 90–95% of all patients having the disease [2]. Patients with T2DM usually have insulin resistance which alters the utilization of endogenously produced insulin at the target cells. During the early stage of the disease, insulin production is increased resulting in hyperinsulinemia. However, as the condition progresses, the production of insulin decreases leading to insulin deficiency [2]. Whereas both type 1 DM and T2DM have a genetic predisposition, the aetiology of T2DM is also related to life style factors such as high fat and sugar intake, physical inactivity and obesity [2]. Worldwide; 346 million people suffer from DM [3] and this disease is ranked as the ninth most common disorder amassing a 68% increase from 1990 to 2010 [4]. Between the years 2010 and 2030, the number of adults with DM in developing countries is expected to increase by 70%, most extensively in Africa [5]. In the Sudan, the prevalence of DM is increasing to epidemic proportions, affecting about 14% of a total population of 31 million [6].

Poorly controlled DM has been associated with increased susceptibility to oral infections including periodontal disease [7–10]. Periodontal disease is characterized by loss of connective tissue and bone support, which eventually might lead to tooth loss. Previous studies have suggested that periodontal infection and DM have a two-way relationship [10,11]. Löe, [12] stated that periodontal disease is the sixth most common complication of DM, whereas Lalla et al., [10] reported that DM is the strongest risk factor for periodontal infection compared to the other systemic conditions such as hypertension. Moreover, it has been demon-

strated that individuals with periodontal pocket ≥6mm are 3.5 times more likely to develop T2DM than those having periodontal pocket <6mm [13]. By now there is strong evidence suggesting that the prevalence and severity of periodontal disease are higher among T2DM patients when compared with non-diabetic individuals [8,9,14–18]. Few studies have reported on no difference in periodontal disease between individuals with and without T2DM [19,20]. Whereas some studies have reported worse periodontal condition among poorly controlled T2DM patients [8,9], others have disconfirmed an association between periodontal disease and metabolic control [21,22].

Few studies have examined the situation of dental caries among T2DM patients. It has been demonstrated that the number of decayed, missed and filled teeth (DMFT) is higher among individuals with than without T2DM [18,23,24]. Moreover, Leung et al., [25] have found the risk of dental caries to be twice as high in T2DM patients compared to healthy controls. Other studies have disconfirmed such an association [26–28]. Compared to coronal caries; a relationship between DM and root surface caries has been more obvious [10]. Thus, individuals with T2DM have higher prevalence of root surface caries compared to non-diabetic individuals [26,29]. This evidence is equivocal as some studies have found no difference in root surface caries between individuals with and without T2DM [28,30].

The overall contribution of oral diseases to quality of life among T2DM patients has not been frequently investigated. According to the literature, oral health-related quality of life (OHRQoL) did not discriminate between individuals with and without DM [31,32]. It has also been reported that patients with both types of DM have limited awareness of the possible health consequences of poor oral health [32]. OHRQoL measures have been tested in various populations to supplement clinical indicators of oral diseases [33]. One promising measure is the oral impact of daily performance inventory (OIDP) [34]. This measure is based on the WHO classification of impairments, disabilities and handicaps [35] and Locker's theoretical framework [36]. Since its development, the OIDP has shown to be reliable and valid in population based studies as well as in studies of patients with specific disorders [37–39]. An Arabic version of the OIDP inventory has been validated and used in the Sudan among 12 years old school children as well as among adult dental attendees with and without mucocutaneous diseases [40–42]. The OIDP has also been applied in other low-income countries such as Tanzania and Uganda [38,39].

In order to control DM in the Sudan, it is important to bridge the gap between physicians and dentists and to increase the involvement of the dental profession in the secondary prevention of this disease. Information about oral manifestations of T2DM should be made easily available to raise the awareness and knowledge of the importance of dental care for T2DM patients. To our knowledge, there is no published data available documenting the oral diseases and self-reported oral health situation among adult diabetic patients in the Sudan. The purpose of this study was to examine the clinical and subjective oral health indicators among T2DM patients receiving ongoing treatment in an outpatient specialist clinic and to compare the observations with age and gender matched non-diabetic controls taken from the same population. It was hypothesized that T2DM patients were more likely to have oral disease and oral impacts on daily performances than non-diabetic controls. A second aim was to - within the T2DM patients- identify clinical and subjective oral health indicators that discriminate between well-controlled and poorly controlled T2DM patients as well as between patients with long and short duration of the disease. It was hypothesized that poorly controlled T2DM patients had more oral disease and more oral impacts on their daily performances than their well-controlled counterparts. Moreover, it was hypothesized that patients with long duration of T2DM had poorer oral health than their counterparts having short duration of the disease.

Materials and Methods

Study design and participants

This study was designed as a gender and age matched case-control study with a ratio of 2 controls per 1 case. Ethical clearance was obtained from the Ministry of Health in the Sudan and from the Norwegian Research Ethical Committee at the University of Bergen (2012/1470/REK vest). Written informed consent was obtained from each participant. The objectives, steps of oral clinical examination and sampling procedures were explained for the participants. All participants were informed about their dental diagnosis and referred for appropriate dental treatment as needed.

Sample size was calculated to be 450 using Openepi version 3.01 with a power of 90%, alpha level of 0.05, ratio of controls to cases of 2, percentage of exposed controls of 50% and an odds ratio (OR) of 2 as a minimum difference between groups to be detected. From July 2012 to December 2012, 157 cases previously diagnosed with T2DM (63 males and 94 females) who attended for dental treatment at Jaber Abol'ez Diabetes Center were invited to participate in the study. A total of 304 non-diabetic controls (119 male and 185 female) were recruited from the outpatient dental clinic at the Khartoum Dental Teaching Hospital. Illegibility criteria for enrolment of the cases were (i), being diagnosed with T2DM for more than one year and attending a specialized diabetes clinic (ii), having at least 10 remaining natural teeth (iii), no antibiotic, no steroidal and/or non-steroidal anti-inflammatory medication used during the last 3 weeks (iv), not treated with immunosuppressive chemotherapy, no current acute illness, no professional periodontal treatment received during the last 6 months and no pregnancy or lactation. The non-diabetic controls were selected according to the same criteria except for being diagnosed with DM. At the time of enrollment; glycosylated haemoglobin test (HbA1c) was undertaken for the T2DM cases to determine the level of glycemic control (well-controlled: HbA1c ≤8% and poorly controlled: HbA1c >8%) [19]. The test was performed at the laboratory of Jaber Abol'ez Diabetes Center using a commercial kit (LabonaCheck™ A1c analyzer). Individuals in the control group were asked about signs and symptoms of DM and if suspected, they were referred to Khartoum Teaching Hospital for confirmation.

Interview

All participants were interviewed by a trained research assistant using a standardized structured questionnaire. The interview schedule was constructed in English, but participants were interviewed in Arabic and some illustrations were used to help the participants to understand the questions. *Socio-demographics* were assessed in terms of age, gender (male, female), educational level and employment status. Educational level was originally measured as (0 = illiterate, 1 = literate, 2 = primary school, 3 = middle school, 4 = high school, 5 = college, 6 = post-graduation studies) and was recoded into illiterate = 1 (including the original category 0) and literate = 2 (including the categories 1–6). Employment status was measured as (0 = unemployed, 1 = student, 2 = housewife, 3 = retired, 4 = employed), then recoded into unemployed = 1 (including the original categories 0–3) and employed = 2 (including the original category 4). *Lifestyle factors*

were assessed in terms of alcohol consumption (yes/no), smoking (yes/no), hypertension (yes/no), regular dental attendance (yes/no) and sugary drinks consumed per day (0 = no, 1 = 1 cup or less, 2 = 2 to 3 cups, 3 = 4 cups or more), recoded into no = 1 (including the original category 0) and yes = 2 (including the categories 1–3). The *self-reported main explanatory variables* were assessed in terms of history of dry mouth (yes/no) and OHRQoL which was assessed using the eight items OIDP frequency inventory; 'During the past 6 months, how often have problems with your mouth and teeth caused you any difficulty with: *eating and chewing food; speaking and pronouncing clearly; cleaning teeth; sleeping and relaxing; smiling and showing teeth without embarrassment; maintaining usual emotional state; carrying out major work and social role; and enjoying contact with people*'. Each item was assessed using a 5-point scale: (1) Never affected; (2) Less than once a month; (3) Once or twice a month; (4) Once or twice a week; (5) Every, or nearly every day. An additive sum score (OIDP ADD) was constructed from the 8 items as originally scored (1–5) (range 8–40). Secondly, each OIDP frequency item was dichotomized, yielding the categories: 0 = never affected (including the original category 1), 1 = affected (including the original categories 2–5). Simple count scores (SC) were created for the OIDP by adding the eight dichotomized variables. For the purpose of cross-tabulation and logistic regression analysis, the OIDP SC scores (0–8) was dichotomized as 0 = no daily performance affected and 1 = at least one daily performance affected. The distribution of the OIDP SC scores supported this cut-off point.

Oral clinical examination

In preparation for the clinical examination, the main single investigator (HGM) was trained and calibrated to perform oral examination and differential diagnosis for dental caries, periodontal disease and other oral disorders at the Department of Clinical Dentistry-University of Bergen, Norway under the supervision of the team's principal investigator (SOI).

For each participant, clinical examination of all teeth (except 3rd molars) and soft tissues of the oral cavity was performed immediately after completion of the interview. Tools used for the examination were (N22) Color Coded Probe 2-4-6-8-10-12 mm markings, (NAB2) Color Coded Nabors Furcation Probe 3-6-9-12 mm markings, curette, mirror, probe, tweezers and cotton rolls. For *plaque index* (PI) [43] and *mobility index* [44], one value was recorded for each tooth (range 0-3). The sum of values of all teeth examined was divided by the number of teeth examined to give the individual scores. PI and mobility index were recoded into low = 1 and high = 2 using the median as a cutoff point for the sub-group analysis. Another dichotomous variable was created for mobility index (mobility index = 0, mobility index ≥1). *Probing depth* (PD) was measured from the gingival margin to the base of the periodontal pocket (mm) at four sites of each tooth (mesial, distal, buccal and lingual). Individual scores were expressed as percentage of sites with PD of ≥4mm. Individuals were diagnosed as having chronic periodontitis if there was at least one site with PD of ≥4mm. *Furcation involvement* (FI) [45] was recorded as grade I (≤3mm), grade II (>3mm) and grade III (through and through). A dichotomous variable for FI was created (FI ≤ grade I, FI ≥ grade II). *Root surface caries* was recorded as (yes/no) and expressed as percentage of teeth with root caries for each individual. *Coronal caries* was measured using DMFT index [46]. Individual scores were calculated as sum of decayed, missed and filled teeth. DMFT was dichotomized into (DMFT = 0, DMFT ≥1). Another cutoff (low: DMFT < median, high: DMFT ≥median) was used for the sub-group analysis.

Statistical analysis

Statistical Package for Social Sciences (SPSS) version 21 was used to analyze the data. The Kappa test was performed to assess the intra-examiner reliability. Chi-square and independent sample T tests were used to assess the differences in categorical and continuous variables between the cases and controls and to identify possible confounding variables. The Cox regression procedure was used to fit conditional logistic regression models since each case was paired with 2 controls. Adjusted odds ratios (ORs) and their 95% confidence intervals (CI) were calculated with T2DM status (cases/controls) as the outcome variable and periodontitis, dental caries and OHRQoL as main explanatory variables whilst adjusted for a number of possible confounding variables (employment status, educational level, consumption of sugary drinks, smoking, hypertension, PI and regular dental attendance). The selection of independents included in the Cox regression model was based on the theoretical relevance and statistical significant relationship with T2DM in the bivariate analysis. Sub-group analysis was performed within the T2DM group to identify the clinical and self-reported oral health indicators that discriminated between the well-controlled (HbA1c ≤8%) and poorly controlled (HbA1c >8%) patients and between patients with long (>10 years) and short (≤10 years) duration of the disease. Two binary logistic regression models were constructed to assess the relationship between mobility of teeth and level of metabolic control and the relationship between DMFT and duration of T2DM. Age, gender and frequency of regular dental attendance were adjusted for during the analyses. *P* values less than 0.05 were considered statistically significant.

Results

Of the 461 individuals recruited for the study, three participants from the T2DM group did not continue with the interview due to time constraints. One participant from the control group was diagnosed with DM and excluded after the recruitment. The mean duration of T2DM was (9±7.4) years. A total of 31.2% of the cases had experience with the diagnosis for more than 10 years, 70% were poorly controlled and 50% had a family history of DM. The oral examination was repeated for 20 participants randomly selected within 2 weeks. Kappa values (k) were 0.74 for tooth mobility, 0.80 for root surface caries, 0.88 for periodontal diagnosis (PD ≥4mm.) and 1.00 for dental caries.

As shown in Table 1, the mean age for both the cases and controls was (52±10.5) years and 39% of both groups were men. A total of 34.4% versus 13.9% ($P<0.001$) of the cases and controls reported any oral impact (OIDP > 0). The two groups differed with respect to chewing problems (22.7% versus 10.9%, $P<0.001$) and sleeping problems (15.6% versus 5.0%, $P<0.001$) (data not shown). Chewing, sleeping, cleaning teeth and smiling were the impacts most frequently mentioned among the cases, whereas chewing and sleeping were those most frequently mentioned among the controls. Reported dry mouth was more common in the cases than the controls (37.7% versus 10.9%, $P<0.001$) (data not shown). Hypertension was also more common in the cases than the controls (31.8% versus 14.9%, $P<0.001$). Visible dental plaque, furcation involvement, tooth mobility, chronic periodontitis, root surface caries and less than 21 remaining teeth were all more frequently observed in the cases group ($P<0.05$).

The Cox regression analysis, adjusting for possible confounding variables, revealed statistically significant covariates of T2DM in terms of tooth mobility (OR = 9.63, 95% CI: 4.29–21.58), furcation involvement (OR = 5.23, 95% CI: 2.79–9.80), chronic periodontitis (OR = 3.97, 95% CI: 2.08–7.59), root surface caries,

Table 1. Distribution of confounders and main explanatory variables according to T2DM status (n = 457).

Confounding factors	Categories	Cases (n = 154)	Controls (n = 303)
Age, mean (SD)		52.6 (10.5)	52.4 (10.5)
Gender, % (n)	Male	39.0 (60)	39.3 (119)
	Female	61.0 (94)	60.7 (184)
Education, % (n)	Illiterate	26.6 (41)	30.7 (93)
	Literate	73.4 (113)	69.3 (210)
Employment status, % (n)	Unemployed	64.9 (100)	63.4 (192)
	Employed	35.1 (54)	36.6 (111)
Consumption of sugary drinks per day, % (n)	No	83.8 (129)	67.7 (205)
	Yes	16.2 (25)	32.3 (98)**
Smoking, % (n)	No	87.0 (134)	81.5 (247)
	Yes	13.0 (20)	18.5 (56)
Hypertension, % (n)	No	68.2 (105)	85.1 (258)
	Yes	31.8 (49)	14.9 (45)**
Regular dental attendance, % (n)	No	92.2 (142)	96.4 (292)
	Yes	7.8 (12)	3.6 (11)
Plaque index, % (n)	Low (< median)	24.0 (37)	63.0 (191)
	High (≥median)	76.0 (117)	37.0 (112)**
Main explanatory variables			
Number of present teeth, % (n)	≤21 teeth	26.0 (40)	9.6 (29)
	> 21 teeth	74.0 (114)	90.4 (274)**
Tooth mobility, % (n)	No	8.4 (13)	46.9 (142)
	Yes	91.6 (141)	53.1 (161)**
Furcation involvement, % (n)	No	55.8 (86)	87.8 (266)
	Yes	44.2 (68)	12.2 (37)**
Chronic periodontitis, % (n)	No	13.6 (21)	45.5 (138)
	Yes	86.4 (133)	54.5 (165)**
Dental caries (DMFT), % (n)	DMFT = 0	5.2 (8)	4.3 (13)
	DMFT> 0	94.8 (146)	95.7 (290)
Root surface caries, % (n)	No	47.4 (73)	60.4 (183)
	Yes	52.6 (81)	39.6 (120)*
OHRQoL, % (n)	OIDP = 0	65.6 (101)	86.1 (261)
	OIDP>0	34.4 (53)	13.9 (42)**

*$P<0.05$.
**$P<0.01$.
doi:10.1371/journal.pone.0082158.t001

(OR = 1.80, 95% CI: 1.07–3.02), OIDP (OR = 3.85, 95% CI: 2.11–7.01), and having more than 21 remaining teeth (OR = 0.34, 95% CI: 0.17–0.68). When all the main explanatory variables were mutually adjusted for, the statistically significant covariates of T2DM were tooth mobility, furcation involvement, chronic periodontitis, OIDP and having more than 21 remaining teeth. The corresponding ORs were 5.90 (95% CI: 2.26–15.39), 2.96 (95% CI: 1.36–6.45), 4.07 (95% CI: 1.74–9.49), 3.46 (95% CI: 1.61–7.42) and 0.23 (95% CI: 0.08–0.63), respectively (Table 2).

Table 3 depicts the differences in socio-demographic characteristics and clinical indicators between the well- and poorly controlled T2DM patients and between patients with long and short duration of the disease. The mean age among short- and long term T2DM cases was (50.72±10.78) and (56.69±8.61) years respectively, ($P<0.001$). The well- and poorly controlled T2DM groups consisted of 46.7% and 68.6% women ($P<0.05$). Regular dental attendance was reported by 3.8% of the short duration cases versus 16.7% of the long duration cases, ($P<0.05$). High mobility index was less prevalent in the well- than the poorly controlled T2DM cases (37.8% versus 59.0%, $P<0.05$), and high median DMFT index was less frequently found in the short- compared with the long duration group (57.5% versus 75.0%, $P<0.05$). More than 21 remaining teeth were more frequently observed in patients with short- than long duration of T2DM (79.2% versus 62.5%, $P<0.05$).

The multiple logistic regression analyses adjusting for the confounding effect of age, gender and dental attendance, revealed that compared to the short duration-T2DM cases, T2DM cases with long duration were more likely to have DMFT (i.e. above the median) with OR of 2.94 (95% CI: 1.24–6.94) (Table 4). Compared to the well-controlled T2DM cases, the poorly controlled counterparts were more likely to be above the median mobility index with OR of 2.60 (95% CI: 1.21–5.55) (Table 5).

Table 2. All main explanatory variables according to T2DM status adjusted for possible confounding variables, (n = 447).

Variables	n	Adjusted OR (95% CI)[a]	Adjusted OR (95% CI)[b]
Number of present teeth			
≤21 teeth	66	1	1
> 21 teeth	381	0.34 (0.17–0.68)**	0.23 (0.08–0.63)**
Tooth mobility			
No	153	1	1
Yes	294	9.63 (4.29–21.58)**	5.90 (2.26–15.39)**
Furcation involvement			
No	344	1	1
Yes	103	5.23 (2.79–9.80)**	2.96 (1.36–6.45)**
Chronic periodontitis			
No	157	1	1
Yes	290	3.97 (2.08–7.59)**	4.07 (1.74–9.49)**
Dental caries (DMFT)			
DMFT = 0	21	1	1
DMFT > 0	426	0.82 (0.28–2.37)	0.38 (0.09–1.66)
Root surface caries			
No	252	1	1
Yes	195	1.80 (1.07–3.02)*	1.65 (0.84–3.26)
OHRQoL			
OIDP = 0	353	1	1
OIDP>0	94	3.85 (2.11–7.01)**	3.46 (1.61–7.42)**

*$P<0.05$.
**$P<0.01$.
a) Adjusted for employment status, educational level, consumption of sugary drinks, hypertension, smoking, plaque index and regular dental attendance.
b) Adjusted for employment status, educational level, consumption of sugary drinks, hypertension, smoking, plaque index, regular dental attendance and other main explanatory variables.
doi:10.1371/journal.pone.0082158.t002

Discussion

The present study confirmed the hypothesis that clinical indicators of periodontal disease, furcation involvement, mobility of teeth and number of teeth present as well as the OIDP discriminated between T2DM patients and their non-diabetic controls. The hypothesis that poorly controlled and long duration-T2DM patients presented with more oral disease and OIDP compared with their well-controlled and short duration counterparts was partly confirmed in this study. Thus, mobility index and dental caries were the only clinical indicators that discriminated significantly between the sub-groups of T2DM cases.

Before discussing the findings further, it is appropriate to consider the limitations and strengths of the study. As both the cases and controls were attending oral health care services for dental treatment, they probably presented with more severe oral diseases and treatment needs compared to their non-dental attendee counterparts. It would have been an advantage to identify mild oral problems representing early signs of T2DM so that the dentist could contribute with referrals of potentially diseased patients. However, the present findings were restricted to subjects already diagnosed with T2DM. Subjects in the control group were asked about signs and symptoms of T2DM. A more appropriate screening of unidentified individuals with T2DM among the controls as suggested by Borrell et al., [47] would have contributed to the internal validity of the results. Although the control group may represent dental attendees in the general Khartoum population, it is possible that the recruitment procedure of the diabetic patients introduced a bias since a convenience sample attending the Jaber Abol'ez Diabetes Center was utilized [48]. Nevertheless, the socioeconomic status did not differ between the cases and controls in this study. The purpose was to identify covariates of T2DM rather than to estimate disease prevalence in the Sudanese population in general.

Recall bias is of major concern when a case-control design is utilized. One advantage of this study includes the verification of information gathered from the study participants by comparing with medical records from the registry, thus reducing the risk of recall- and social desirability bias. The use of the OIDP index with a long history of validation in various contexts and the availability of novel clinical measurements are further strengths. Moreover, many potential confounding factors were considered in the multivariable analysis [49]. This was considered necessary as periodontal disease, dental caries and T2DM are multifactorial diseases.

Consistent with previous studies, the T2DM patients presented with more visible dental plaque, more missing teeth and were more likely to suffer from chronic periodontitis compared with the non-diabetic controls [17,19]. Although the T2DM patients presented with limited sugary drink consumption and low frequency of smoking, PI was significantly higher among the cases than the controls. Presence of more dental plaque and poorer oral hygiene among diabetic compared to non-diabetic subjects have been reported in a number of studies and might be attributed to DM patients having higher levels of glucose in gingival crevicular fluid (GCF) and saliva [8,19]. The prevalence of poor oral hygiene

Table 3. Clinical and self-reported oral health indicators by subgroups of T2DM cases according to glycemic control and duration of diabetes.

Confounding factors	Well-controlled DM: HbA1c ≤8 (n = 45)	Poorly controlled DM: HbA1c > 8 (n = 105)	Short duration: DM ≤10 years (n = 106)	Long duration: DM > 10 years (n = 48)
Age, mean (SD)	53.84 (9.34)	52.13 (10.90)	50.72 (10.78)	56.69 (8.61)**
Gender, % (n)				
Male	53.3 (24)	31.4 (33)	37.7 (40)	41.7 (20)
Female	46.7 (21)	68.6 (72)*	62.3 (66)	58.3 (28)
Education, % (n)				
Illiterate	22.2 (10)	29.5 (31)	29.2 (31)	20.8 (10)
Literate	77.8 (35)	70.5 (74)	70.8 (75)	79.2 (38)
Employment status, % (n)				
Unemployed	51.1 (23)	71.4 (75)	61.3 (65)	72.9 (35)
Employed	48.9 (22)	28.6 (30)*	38.7 (41)	27.1 (13)
Regular dental attendance, % (n)				
No	93.3 (42)	91.4 (96)	96.2 (102)	83.3 (40)
Yes	6.7 (3)	8.6 (9)	3.8 (4)	16.7 (8)*
Plaque index, mean (SD)	1.62 (0.41)	1.64 (0.33)	1.62 (0.34)	1.65 (0.37)
**Main explanatory variables**				
Tooth mobility, % (n)				
Low (< median)	62.2 (28)	41.0 (43)	50.9 (54)	41.7 (20)
High (≥median)	37.8 (17)	59.0 (62)*	49.1 (52)	58.3 (28)
Chronic periodontitis, % (n)				
No	15.6 (7)	12.4 (13)	11.3 (12)	18.8 (9)
Yes	84.4 (38)	87.6 (92)	88.7 (94)	81.2 (39)
Furcation involvement, % (n)				
No	64.4 (29)	51.4 (54)	57.5 (61)	52.1 (25)
Yes	35.6 (16)	48.6 (51)	42.5 (45)	47.9 (23)
Root surface caries, % (n)				
No	51.1 (23)	45.7 (48)	52.8 (56)	35.4 (17)
Yes	48.9 (22)	54.3 (57)	47.2 (50)	64.6 (31)
Dental caries (DMFT), % (n)				
Low (< median)	42.2 (19)	34.3 (36)	42.5 (45)	25.0 (12)
High (≥median)	57.8 (26)	65.7 (69)	57.5 (61)	75.0 (36)*
Number of present teeth, % (n)				
≤21 teeth	31.1 (14)	22.9 (24)	20.8 (22)	37.5 (18)
> 21 teeth	68.9 (31)	77.1 (81)	79.2 (84)	62.5 (30)*
OHRQoL, % (n)				
OIDP = 0	73.3 (33)	63.8 (67)	69.8 (74)	56.2 (27)
OIDP>0	26.7 (12)	36.2 (38)	30.2 (32)	43.8 (21)

*$P<0.05$.
**$P<0.01$.
doi:10.1371/journal.pone.0082158.t003

among dental attendees from the general population in Khartoum has been reported to be about 39% [50]. This figure is consistent with that of 37% observed in the control group of the present study, suggesting that this group mirrors the background population as well. More missing teeth among the T2DM patients has been reported to be a characteristic of a population with poor oral hygiene [18]. Corresponding difference has not been disclosed in populations with good oral hygiene [51].

Since the relationship between T2DM and periodontal disease have been investigated in few countries [52], this study emanating from sub-Saharan Africa provides a valuable contribution to the literature. Notably, the prevalence of chronic periodontitis as defined by pocket depths rather than clinical attachment level might have been underestimated [53]. Moreover, the lack of a generally accepted case definition throughout studies impedes comparison of prevalence figures between them [54]. In addition

Table 4. DMFT regressed on duration of T2DM; adjusted for age, gender and regular dental attendance.

Variables	n	OR (95% CI)
Age	153	0.99 (0.95–1.02)
Gender		
Male	59	1
Female	94	3.45 (1.67–7.14)**
Regular dental attendance		
No	141	1
Yes	12	0.57 (0.15–2.18)
Duration of DM		
DM ≤10 years	105	1
DM > 10 years	48	2.94 (1.24–6.94)*

*$P<0.05$.
**$P<0.01$.
-2 Log likelihood = 183.536, Cox & Snell $R^2 = 0.114$, Nagelkerke $R^2 = 0.155$.
doi:10.1371/journal.pone.0082158.t004

to chronic periodontitis per se, clinical signs such as tooth mobility and furcation involvement were more frequent among the cases compared to their non-diabetic controls. These results coincide with findings from previous studies [16,18]. Although the mechanism by which DM affects periodontal tissues is not fully understood [55], it has been suggested that the magnitude of inflammatory response of periodontal tissues to oral micro-flora is exaggerated in DM patients. Moreover, advanced glycation end products (AGEs) are formed when excess glucose comes in contact with specific proteins which triggers series of pro-inflammatory reactions [56,57].

Although the T2DM patients in this study presented with more visible dental plaque and more missing teeth than non-diabetic subjects, there was no difference with respect to DMFT status. In this aspect, the present findings corroborate with those reported by other researchers [26,51,58]. A lack of a significant relationship between DM and dental caries has been attributed to confounding factors such as xerostomia, periodontal disease and a strict carbohydrate diet [59]. Nevertheless, a recent study by Jawed et al., [23] reported higher levels of DMFT among individuals with than without T2DM. An important finding in this study is that both the cases and controls presented with high prevalence of DMFT (95%), reflecting an urgent need for treatment and preventive oral health care programs. Whereas Hintao et al., [26] found a significant higher prevalence of root surface caries among T2DM patients compared to non-diabetic controls, the present finding suggesting absence of such an association corroborates those reported by Collin et al., [28] and Lin et al., [30]. The importance of measuring psychological and social impacts of oral diseases as a supplement to clinical indicators was pointed out by Cohen and Jago [33]. In this study, the T2DM patients were almost 3 times more likely than their non-diabetic controls to have at least one oral impact on their daily performances. This is at odds with the results of other studies suggesting that oral problems might not be a priority in DM subjects who are burdened with health complications in general [31,32].

The majority (70%) of the T2DM patients investigated in this study were poorly controlled. This agrees with recent data from other studies and illustrates the difficulties in maintaining good glycemic control [60]. In the present study, the poorly controlled

Table 5. Mobility index regressed on the level of glycemic control; adjusted for age and gender.

Variables	n	OR (95% CI)
Age	149	1.05 (1.01–1.08)**
Gender		
Male	56	1
Female	93	1.32 (0.63–2.78)
Glycemic control		
Well-controlled DM	45	1
Poorly controlled DM	104	2.60 (1.21–5.55)*

*$P<0.05$.
**$P<0.01$.
-2 Log likelihood = 191.545, Cox & Snell $R^2 = 0.093$, Nagelkerke $R^2 = 0.124$.
doi:10.1371/journal.pone.0082158.t005

cases were 2.6 times more likely to present with high mobility index (Table 5). This is consistent with other studies reporting a significant worse periodontal condition among poorly controlled DM patients compared to patients with good metabolic control [7–10].

Duration of DM might play an important role when the relation between DM and oral diseases is investigated [61]. The level of coronal caries was significantly higher in the long- compared to the short duration-T2DM group. This finding is in agreement with the normal pathogenesis of dental caries as "time" is an important factor for the development of the disease [62]. Moreover, the long duration-T2DM group had fewer teeth, which corroborates with the study of Santos et al., [21] in which the number of remaining teeth was negatively correlated with HbA1c levels.

Conclusion

This study revealed that chronic periodontitis, tooth mobility, furcation involvement and OIDP were more prevalent among T2DM patients than their non-diabetic matched controls. Although pocket depths as a measure of chronic periodontitis did not associate with the level of glycemic control or the duration of T2DM, there was an association in the expected direction with tooth mobility and dental caries. The present findings have implications for both DM and oral health care provision. For future prevention and management, it is important to know whether periodontitis plays a role in the development and control of DM and its complications [63]. Further large scale prospective studies are needed to investigate the effect of treatment of periodontitis on the management and control of T2DM.

Acknowledgments

Authors are grateful to Dr. Alrashid Abdulla, the Director of Jaber Abol'ez Diabetes Center, and to other staff members at Jaber Abol'ez Diabetes Center, Faculty of Dentistry- University of Khartoum and Khartoum Dental Teaching Hospital for logistic support. We acknowledge all diabetic and non-diabetic patients, as well as dental assistants who helped in data collection and made this work possible.

Author Contributions

Conceived and designed the experiments: HGM MFA SOI ANÅ. Performed the experiments: HGM SBI. Analyzed the data: OEB ANÅ. HGM. Contributed reagents/materials/analysis tools: SOI HGM KM. Wrote the paper: HGM ANÅ SOI.

References

1. Alberti KG, Zimmet PZ (1998) Definition, diagnosis and classification of diabetes mellitus and its complications. Part 1: diagnosis and classification of diabetes mellitus provisional report of a WHO consultation. Diabet Med 15: 539–553.
2. Mealey BL, Ocampo GL (2007) Diabetes mellitus and periodontal disease. Periodontol 2000 44: 127–153.
3. (Aug, 2011) WHO fact sheet, Diabetes.
4. Vos T, Flaxman AD, Naghavi M, Lozano R, Michaud C, et al. (2012) Years lived with disability (YLDs) for 1160 sequelae of 289 diseases and injuries 1990–2010: a systematic analysis for the Global Burden of Disease Study 2010. Lancet 380: 2163–2196.
5. Shaw JE, Sicree RA, Zimmet PZ (2010) Global estimates of the prevalence of diabetes for 2010 and 2030. Diabetes Research and Clinical Practice 87: 4–14.
6. Abdelgadir M, Elbagir M, Eltom M, Berne C (2006 Oct) The influence of glucose self-monitoring on glycaemic control in patients with diabetes mellitus in Sudan. Diabetes Res Clin Pract 74: 90–94.
7. Lim LP, Tay FB, Sum CF, Thai AC (2007 Feb) Relationship between markers of metabolic control and inflammation on severity of periodontal disease in patients with diabetes mellitus. J Clin Periodontol 2007 Feb;34(2):118–23 34: 118–123.
8. Campus G, Salem A, Uzzau S, Baldoni E, Tonolo G (2005 Mar) Diabetes and periodontal disease: a case-control study. J Periodontol 76: 418–425.
9. Tsai C, Hayes C, Taylor GW (2002 Jun) Glycemic control of type 2 diabetes and severe periodontal disease in the US adult population. Community Dent Oral Epidemiol 30: 182–192.
10. Lalla E, Lamster IB (2012) Assessment and management of patients with diabetes mellitus in the dental office. Dent Clin North Am 56: 819–829.
11. Taylor GW (2001 Dec) Bidirectional interrelationships between diabetes and periodontal diseases: an epidemiologic perspective. Ann Periodontol 6: 99–112.
12. Löe H (1993 Jan) Periodontal disease. The sixth complication of diabetes mellitus. Diabetes Care 16: 329–334.
13. Morita I, Inagaki K, Nakamura F, Noguchi T, Matsubara T, et al. (2012 Feb) Relationship between periodontal status and levels of glycated hemoglobin. J Dent Res ;91(2):161–6 91: 161.
14. Susanto H, Nesse W, Dijkstra PU, Agustina D, Vissink A, et al. (2011 Apr) Periodontitis prevalence and severity in Indonesians with type 2 diabetes. J Periodontol 82: 550–557.
15. Preshaw PM, de Silva N, McCracken GI, Fernando DJ, Dalton CF, et al. (2010 Feb) Compromised periodontal status in an urban Sri Lankan population with type 2 diabetes. J Clin Periodontol 37: 165–171.
16. Khader YS, Albashaireh ZS, Hammad MM (2008 May-Jun) Periodontal status of type 2 diabetics compared with nondiabetics in north Jordan. East Mediterr Health J 2008 May-Jun;14(3):654–61 14: 654–661.
17. Susanto H, Nesse W, Dijkstra PU, Hoedemaker E, van Reenen YH, et al. (2012 Aug) Periodontal inflamed surface area and C-reactive protein as predictors of HbA1c: a study in Indonesia. Clin Oral Investig 16: 1237–1242.
18. Tanwir F, Altamash M, Gustafsson A (2009) Effect of diabetes on periodontal status of a population with poor oral health. Acta Odontol Scand 67: 129–133.
19. Ribeiro FV, de Mendonça AC, Santos VR, Bastos MF, Figueiredo LC, et al. (2011 Aug) Cytokines and bone-related factors in systemically healthy patients with chronic periodontitis and patients with type 2 diabetes and chronic periodontitis. J Periodontol 82: 1187–1196.
20. Costa PP, Trevisan GL, Macedo GO, Palioto DB, Souza SL, et al. (2010 Mar) Salivary interleukin-6, matrix metalloproteinase-8, and osteoprotegerin in patients with periodontitis and diabetes. J Periodontol 81: 384–391.
21. Santos VR, Lima JA, Miranda TS, Feres M, Zimmermann GS, et al. (2012 Mar) Relationship between glycemic subsets and generalized chronic periodontitis in type 2 diabetic Brazilian subjects. Arch Oral Biol 57: 293–299.
22. Alpagot T, Silverman S, Lundergan W, Bell C, Chambers DW (2001 Jun) Crevicular fluid elastase levels in relation to periodontitis and metabolic control of diabetes. J Periodontal Res ;36(3):169–74 36: 169–174.
23. Jawed M, Khan RN, Shahid SM, Azhar A (Exp Diabetes Res. 2012;2012:947304) Protective effects of salivary factors in dental caries in diabetic patients of Pakistan.
24. Jawed M, Shahid SM, Qader SA, Azhar A (2011 May-Jun) Dental caries in diabetes mellitus: role of salivary flow rate and minerals. J Diabetes Complications ;25(3):183–6 25: 183–186.
25. Leung WK, Siu SC, Chu FC, Wong KW, Jin L, et al. (2008) Oral health status of low-income, middle-aged to elderly Hong Kong Chinese with type 2 diabetes mellitus. Oral Health Prev Dent 6: 105–118.
26. Hintao J, Teanpaisan R, Chongsuvivatwong V, Dahlen G, Rattarasarn C (2007 Aug) Root surface and coronal caries in adults with type 2 diabetes mellitus. Community Dent Oral Epidemiol 35: 302–309.
27. Zielinski MB, Fedele D, Forman LJ, Pomerantz SC (2002 May-Jun) Oral health in the elderly with non-insulin-dependent diabetes mellitus. Spec Care Dentist 2002 May-Jun;22(3):94–8 22: 94–98.
28. Collin HL, Uusitupa M, Niskanen L, Koivisto AM, Markkanen H, et al. (1998 Jun) Caries in patients with non-insulin-dependent diabetes mellitus. Oral Surg Oral Med Oral Pathol Oral Radiol Endod ;85(6):680–5 85: 680–685.
29. Hintao J, Teanpaisan R, Chongsuvivatwong V, Ratarasan C, Dahlen G (2007 Jun) The microbiological profiles of saliva, supragingival and subgingival plaque and dental caries in adults with and without type 2 diabetes mellitus. Oral Microbiol Immunol 22: 175–181.
30. Lin BP, Taylor GW, Allen DJ, Ship JA (1999 Jan-Feb) Dental caries in older adults with diabetes mellitus. Spec Care Dentist ;19(1):8–14 19: 8–14.
31. Busato IM, Ignácio SA, Brancher JA, Moysés ST, Azevedo-Alanis LR (2012 Feb) Impact of clinical status and salivary conditions on xerostomia and oral health-related quality of life of adolescents with type 1 diabetes mellitus. Community Dent Oral Epidemiol 40: 62–69.
32. Allen EM, Ziada HM, O'Halloran D, Clerehugh V, Allen PF (2008 Mar) Attitudes, awareness and oral health-related quality of life in patients with diabetes. J Oral Rehabil 35: 218–223.
33. Cohen LK, Jago JD (1976) Toward the formulation of sociodental indicators. Int J Health Serv 6: 681–698.
34. Adulyanon S, Sheiham A (1997) Oral Impact on Daily Performances. In Measuring Oral Health and Quality of Life. Edited by Slade GD. North Carolina: Department of Dental Ecology, School of Dentistry, University of North Carolina. 152–160.
35. World Health Organisation: International classification of impairments, disabilities and handicaps. Geneva: World Health Organisation 1980.
36. Locker D (1988 Mar) Measuring oral health: a conceptual framework. Community Dent Health 5: 3–18.
37. Allen PF (2003 Sep 8) Assessment of oral health related quality of life. Health Qual Life Outcomes ;1:40 1:40.
38. Astrøm AN, Okullo I (2003 Aug 28) Validity and reliability of the Oral Impacts on Daily Performance (OIDP) frequency scale: a cross-sectional study of adolescents in Uganda. BMC Oral Health ;3(1):5 3: 5.
39. Masalu JR, Astrøm AN (2003 Feb) Applicability of an abbreviated version of the oral impacts on daily performances (OIDP) scale for use among Tanzanian students. Community Dent Oral Epidemiol 31: 7–14.
40. Nurelhuda NM, Ahmed MF, Trovik TA, Åstrøm AN (2010 Dec 23) Evaluation of oral health-related quality of life among Sudanese schoolchildren using Child-OIDP inventory. Health Qual Life Outcomes 8:152.
41. Ali WM Assessment of oral impact on daily performance, dental caries, oral hygiene and periodontal disease among a group of adult dental patients Master thesis of Philosophy in Oral sciences.: University of Bergen; 2011.
42. Suliman NM, Johannessen AC, Ali RW, Salman H, Astrøm AN (2012 Jul 8) Influence of oral mucosal lesions and oral symptoms on oral health related quality of life in dermatological patients: a cross sectional study in Sudan. BMC Oral Health 12:19.
43. Silness J, Loe H (1964) Periodontal Disease In Pregnancy. Ii. Correlation Between Oral Hygiene And Periodontal Condtion. Acta Odontol Scand 22: 121–135.
44. Miller SC (1938) Textbook of Periodontia: Philadelphia, Blakiston.
45. Hamp SE, Nyman S, Lindhe J (1975) Periodontal treatment of multirooted teeth. Results after 5 years. J Clin Periodontol 2: 126–135.
46. World Health Organization: Oral Health Surveys, Basic Methods, ed 4. Geneva, World Health Organization, 1997.
47. Borrell LN, Kunzel C, Lamster I, Lalla E (2007) Diabetes in the dental office: using NHANES III to estimate the probability of undiagnosed disease. J Periodontal Res 42: 559–565.
48. Vandenbroucke JP, von Elm E, Altman DG, Gotzsche PC, Mulrow CD, et al. (2007) Strengthening the Reporting of Observational Studies in Epidemiology (STROBE): explanation and elaboration. Epidemiology 18: 805–835.
49. Bjelland S, Bray P, Gupta N, Hirscht R (2002) Dentists, diabetes and periodontitis. Aust Dent J 47: 202–207; quiz 272.
50. Nasir EF, Astrom AN, David J, Ali RW (2009) Utilization of dental health care services in context of the HIV epidemic- a cross-sectional study of dental patients in the Sudan. BMC Oral Health 9: 30.
51. Sandberg GE, Sundberg HE, Fjellstrom CA, Wikblad KF (2000 Sep) Type 2 diabetes and oral health: a comparison between diabetic and non-diabetic subjects. Diabetes Res Clin Pract 2000 Sep;50(1):27–34 50: 27–34.
52. Borgnakke WS, Ylostalo PV, Taylor GW, Genco RJ (2013) Effect of periodontal disease on diabetes: systematic review of epidemiologic observational evidence. J Clin Periodontol 40 Suppl 14: S135–152.
53. Albandar JM, Muranga MB, Rams TE (2002) Prevalence of aggressive periodontitis in school attendees in Uganda. J Clin Periodontol 29: 823–831.
54. Leroy R, Eaton KA, Savage A (2010) Methodological issues in epidemiological studies of periodontitis--how can it be improved? BMC Oral Health 10: 8.
55. Nassar H, Kantarci A, van Dyke TE (2007) Diabetic periodontitis: a model for activated innate immunity and impaired resolution of inflammation. Periodontol 2000 43: 233–244.
56. Lamster IB, Lalla E, Borgnakke WS, Taylor GW (2008 Oct) The relationship between oral health and diabetes mellitus. J Am Dent Assoc 139: 19S–24S.
57. Takeda M, Ojima M, Yoshioka H, Inaba H, Kogo M, et al. (2006 Jan) Relationship of serum advanced glycation end products with deterioration of periodontitis in type 2 diabetes patients. J Periodontol 77: 15–20.
58. Arrieta-Blanco JJ, Bartolomé-Villar B, Jiménez-Martinez E, Saavedra-Vallejo P, Arrieta–Blanco FJ (2003 Mar-Apr) Bucco-dental problems in patients with Diabetes Mellitus (I) : Index of plaque and dental caries. Med Oral 8: 97–109.

59. Moore PA, Guggenheimer J, Etzel KR, Weyant RJ, Orchard T (2001 Sep) Type 1 diabetes mellitus, xerostomia, and salivary flow rates. Oral Surg Oral Med Oral Pathol Oral Radiol Endod 92: 281–291.
60. Hodge PJ, Robertson D, Paterson K, Smith GL, Creanor S, et al. (2012) Periodontitis in non-smoking type 1 diabetic adults: a cross-sectional study. J Clin Periodontol 39: 20–29.
61. Moore PA, Weyant RJ, Mongelluzzo MB, Myers DE, Rossie K, et al. (1999) Type 1 diabetes mellitus and oral health: assessment of periodontal disease. J Periodontol 70: 409–417.
62. Selwitz RH, Ismail AI, Pitts NB (2007) Dental caries. The Lancet 369: 51–59.
63. Lalla E, Papapanou PN (2011) Diabetes mellitus and periodontitis: a tale of two common interrelated diseases. Nat Rev Endocrinol 7: 738–748.
